# Intravascular ultrasound findings and stent implantation for a patient with coronary spastic angina at site of progressive atherosclerotic plaque and responded poorly to medical treatment: a case report

**DOI:** 10.1186/s12872-019-01304-3

**Published:** 2019-12-17

**Authors:** Haoran Wang, Geng Peng, Yancai Dong, Dongliang Liu

**Affiliations:** Cardiovascular Institute of Luohe and Department of Cardiology, Luohe Central Hospital, Luohe Medical College, 56# Renmin Ave., Luohe, 462000 People’s Republic of China

**Keywords:** Coronary spastic angina, Intravascular ultrasound, Atherosclerotic plaque, Stenting

## Abstract

**Background:**

Most coronary spastic angina patients are responsive to coronary vasodilators therapy, and stent implantation is not recommended for regular use. We reported the angiographic and intravascular ultrasound (IVUS) images of a rare case who responded poorly to medical treatment due to progressive atherosclerotic plaque at the spastic site.

**Case presentation:**

A 60-year-old man complaining of 1-month history of episodic chest pain at rest was admitted to our hospital. The diagnosis of coronary spastic angina was made based on the angiographic evidence of vasospasm at the right coronary artery (RCA). The patient responded poorly to conventional medical treatment during the 1-year follow-up. The repeated angiography revealed totally occlusion of the proximal segment of the RCA at the same location as 1 year before, and IVUS demonstrated there was vulnerable plaque and thrombus at the site of spasm. Episodic chest pain ceased completely in the follow up period after stenting.

**Conclusion:**

Coronary spasm might present at the vessel site with advanced atherosclerotic plaque. For patients with refractory vasospastic angina and significant occlusion, stenting might be a viable and valuable treatment strategy under the guidance of intracoronary imaging.

## Background

Coronary artery vasospasm plays an important role in the pathogenesis of acute coronary syndrome and ventricular arrhythmia, leading to myocardial infarction, ventricular tachycardia or sudden death [1]. Previous studies suggest that spasm is due to the transient abnormal or hypersensitive response of the lesion segment to various stimuli [2]. Despite this, the pathohistological and morphological features of culprit coronary segments have not been fully understood. According to the reports of angiographic images, coronary spasm is likely to occur at the site of early stages of atherosclerosis rather than advanced atherosclerosis [3, 4]. Most patients are responsive to coronary vasodilators therapy (calcium channel antagonists and nitrates), and stent implantation is not recommended for regular use [5, 6]. Unlike previous studies, the current study reports the angiographic and intravascular ultrasound (IVUS) images of a patient with coronary artery spasm at site of progressive atherosclerotic plaque and responded poorly to medical treatment. The episodic angina attack was released completely after the implantation of stents.

## Case presentation

The current case was reported according to The CARE guidelines: consensus-based clinical case reporting guideline development [7]. Timeline of relevant events were presented in Table 1.

**Table 1:** Timeline of relevant events

Time	Events
15-Jul-16	Onset of chest pain
23-Aug-16	First hospitalization
23-Aug-16	First angiography
1-Apr-17	Monitor ECG
14-Jul-17	Second hospitalization
18-Jul-17	Second angiography and IVUS
8-Oct-18	Follow up

**Fig. 1:**
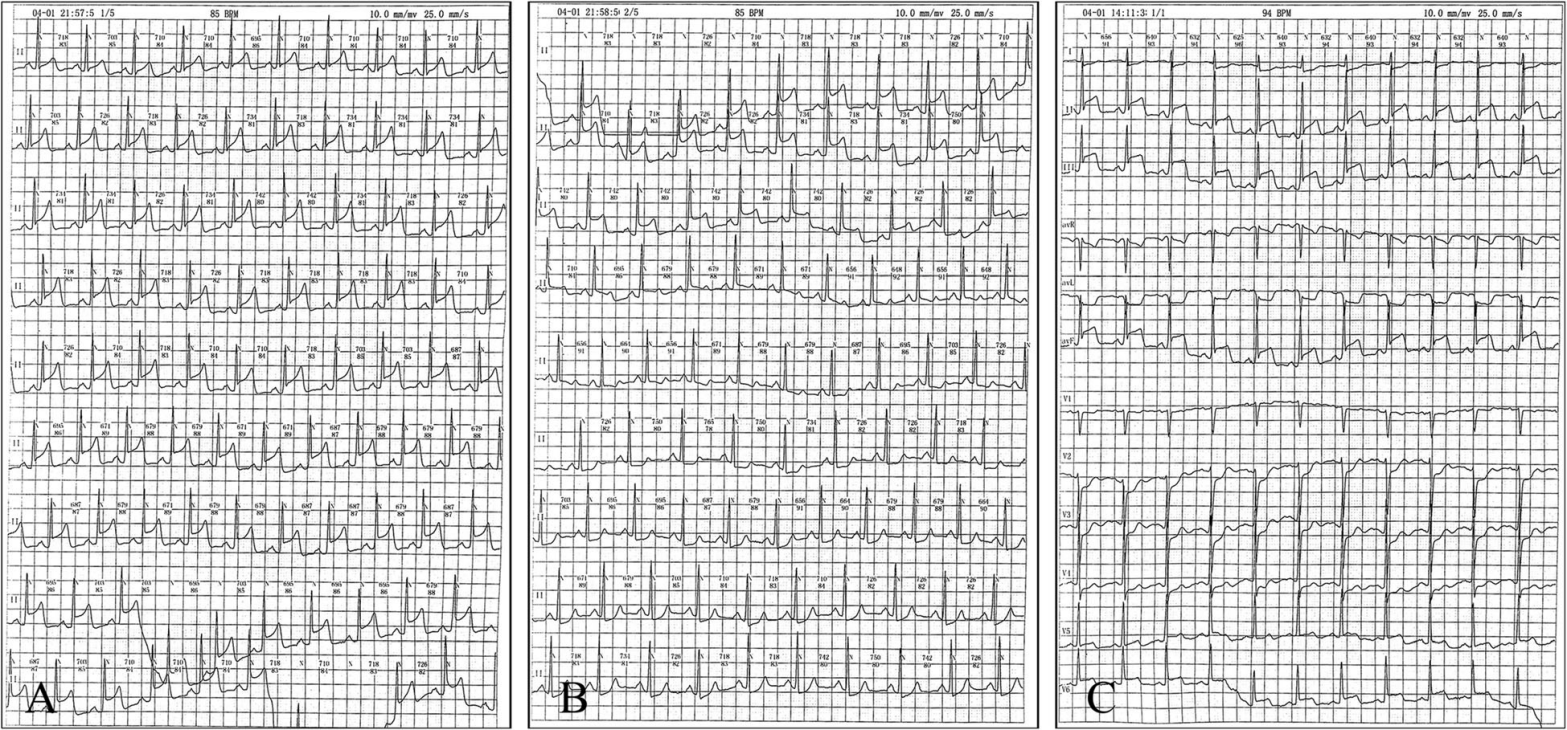
Holter records during the chest pain attack. **a** and **b**: sequential change of lead II during an entire episode of chest pain; **c**: 12-leads ECG recorded during chest pain showed ST-segment elevation in leads II, III and avF

**Fig. 2:**
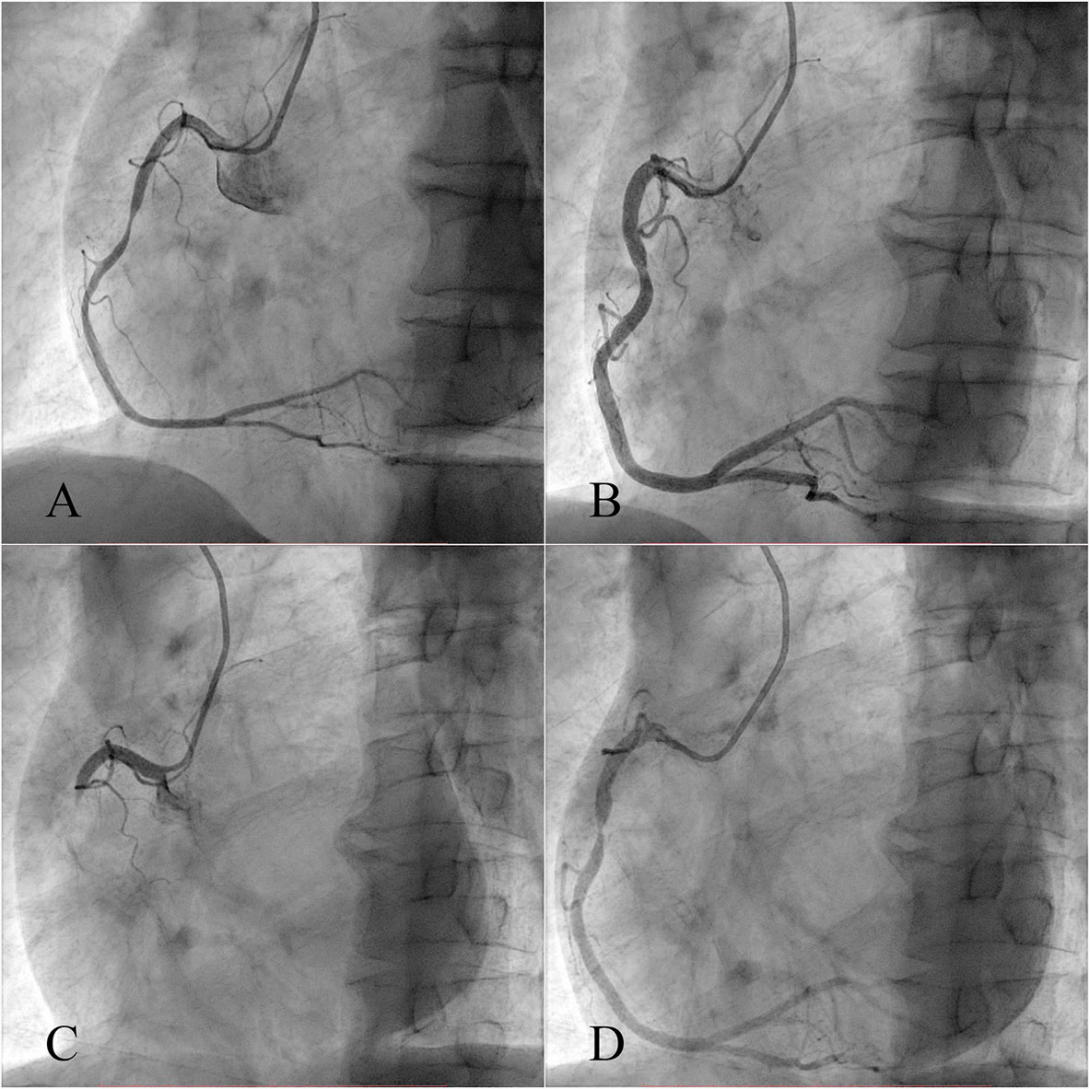
Coronary angiography of the RCA. **a**: at presentation, there was severe nonobstructive coronary stenosis (about 90%) in the right coronary artery; **b**: an intracoronary injection of nitroglycerin via the catheter improved the occlusion; **c**: at follow-up, coronary angiography revealed totally occlusion of the proximal segment of the RCA at the same location as one year before; **d**: an intracoronary injection of nitroglycerin via the catheter restored the blood flow, and significant stenosis (about 95%) was observed

**Fig. 3:**
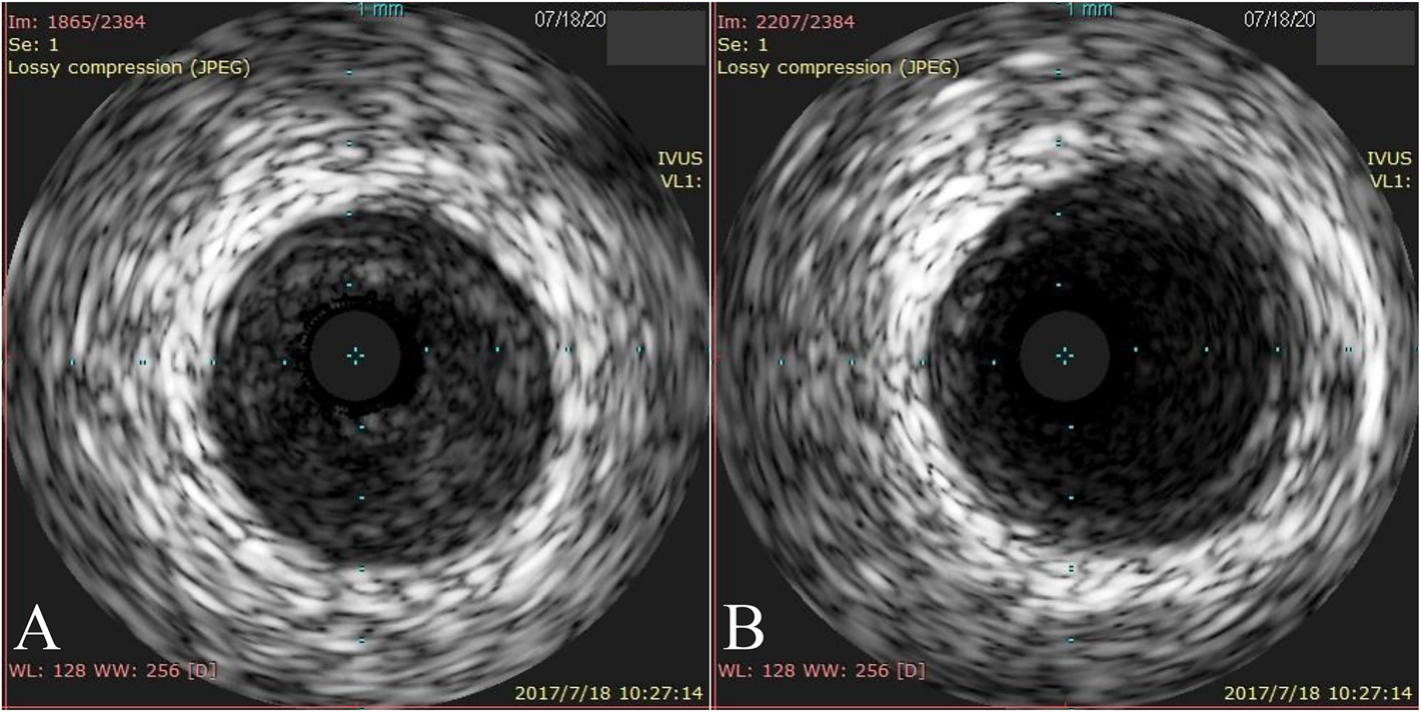
IVUS findings of the right coronary artery around the spastic segment. **a**: at the site of spasm, the minimal lumen area was 2.26 mm^2^; **b**: the vessel size of distal reference segment in RCA was about 13.47 mm^2^


A 60-year-old man complaining of 1-month history of episodic chest pain at rest was admitted to our hospital. He had a smoking history of 1 pack/day for 30 years. The episodes occurred 3–5 times per day while the patient was at rest, lasted for 1–3 min, and resolved without intervention. The monitor electrocardiogram (ECG) demonstrated ST segment elevation in leads II, III, and aVF with reciprocal ST segment depression in the anterior leads during the episodes of angina (Fig. 1). Findings from a troponin I test and echocardiography were normal. Cardiac catheterization was performed, as shown in Fig. 2a and Additional file 1: Video S1, there was severe nonobstructive coronary stenosis (about 90%) in the right coronary artery (RCA). An intracoronary injection of nitroglycerin via the catheter improved the occlusion of his RCA (Fig. 2b and Additional file 2: Video S2). A diagnosis of coronary spastic angina was made. A high dose calcium channel blocker (diltiazem 90 mg bid) and long acting nitrates were prescribed (isosorbide dinitrate 50 mg qd). Other medical treatment including aspirin 100 mg qd, clopidogrel 75 mg qd, atorvastatin 20 mg qd and imidapril 10 mg qd. During the 1-year follow-up period, he reported that his angina responded poorly to the medicine. ST segment elevation in leads II, III, and aVF during the episodes of angina was recorded by the monitor ECG. The patient underwent a repeated coronary angiography which revealed totally occlusion of the proximal segment of the RCA at the same location as one year before (Fig. 2c and Additional file 3: Video S3). An intracoronary injection of nitroglycerin via the catheter restored the blood flow with significant stenosis (about 95%) observed (Fig. 2d and Additional file 4: Video S4). IVUS found there was diffuse low echogenic plaque around the spastic site, characterized by thin fibrous cap overlying a lipid-rich plaque, with erosion, rupture and formation of small thrombosis (Additional file 5: Video S5), which indicated a combination of spasm and typical myocardial infarction pathophysiology [8]. The minimal lumen area was 2.26 mm^2^ (Fig. 3a) and the vessel size of reference segment was about 13.41 mm^2^ (Fig. 3b). We placed two stents in the RCA to stabilize this culprit lesion. Immediate result was satisfactory on angiography and IVUS evaluation (Additional file 6: Video S6 and Additional file 7: Video S7). More importantly, episodic chest pain ceased completely in the follow up period.

**Additional file 1: Video S1.** At presentation, there was severe nonobstructive coronary stenosis (about 90%) in the right coronary artery.



**Additional file 2: Video S2.** An intracoronary injection of nitroglycerin via the catheter improved the occlusion.



**Additional file 3: Video S3.** At follow-up, coronary angiography revealed totally occlusion of the proximal segment of the RCA at the same location as 1 year before.



**Additional file 4: Video S4.** An intracoronary injection of nitroglycerin via the catheter restored the blood flow, and significant stenosis (about 95%) was observed.



**Additional file 5: Video S5.** Intravascular ultrasound indicated there was diffuse low echogenic plaque without significant calcification around the spastic site.


## Discussion and conclusion

The current study reported an atypical case of coronary vasospastic angina. A patient suffered from recurrent and refractory RCA spasm who eventually presented with a combination of spasm and typical myocardial infarction pathophysiology, and was treated with stent implantation. The IVUS shows that there was diffuse low echogenic plaque around the spastic site, characterized by thin fibrous cap overlying a lipid-rich plaque, with erosion, rupture and small thrombosis formation. The episodic angina attack was released completely after the implantation of stents.

The unique characteristics of the current case includes the advanced atherosclerotic plaque presented at the site of spasm, the resistance to calcium channel inhibitors and nitrates, and the satisfactory effectiveness achieved after stents implantation.

In accordance with the current case, other studies also reported plaque erosion at the site of coronary spasm, which indicates that intracoronary imaging can help to diagnose more complex cases at which standard coronary angiogram does not identify the possible problem or culprit [9–11]. And stent placement under the guidance of intracoronary imaging represents an attractive therapeutic option in patients with vasospastic angina refractory to aggressive medical therapy [12, 13].

## Supplementary information

**Additional file 6: Video S6.** Two stents were placed in the RCA and immediate result was satisfactory on angiography.

**Additional file 7: Video S7.** Intravascular ultrasound indicated the stents were expanded well. The minimum stent area was 11.22mm^2^, minimum stent diameter was 3.62 mm, maximum stent diameter was 4.12 mm, stent expansion was 83.67%.

## Data Availability

All relevant data supporting the conclusions of this article are included within the article.

## Abbreviations

ECG: Electrocardiogram
IVUS: Intravascular ultrasound
RCA: Right coronary artery
